# miRNA expression in human lung cancer and fetal lung: a comparative study

**DOI:** 10.1186/1753-6561-7-S2-P67

**Published:** 2013-04-04

**Authors:** Daiana D Becker-Santos, Kelsie L Thu, Patricia P Reis, Wendy P Robinson, Stephen Lam, Wan L Lam

**Affiliations:** 1 Department of Integrative Oncology, British Columbia Cancer Research Centre, Vancouver, BC, Canada; 2 Interdisciplinary Oncology Program, University of British Columbia, Vancouver, BC, Canada; 3 Department of Surgery and Orthopaedics, Faculty of Medicine, São Paulo State University, UNESP, Botucatu, SP, Brazil; 4 Department of Medical Genetics, University of British Columbia, Vancouver, BC, Canada

## Background

Many molecular systems involved in controlling development are also implicated in malignant transformation and are associated with poorer survival outcomes in cancer patients. In fact, lung tumours with similar gene expression profiles to fetal lung cells are associated with aggressive phenotypes of the disease. As targeting fetal developmental pathways selectively reactivated in cancer cells would presumably spare normal adult cells from toxicity, these pathways are considered ideal therapeutic targets for cancer treatment. Therefore, a better understanding of the interplay between fetal lung development and lung cancer on the level of individual miRNAs will provide new insights for translational research. In this study we applied sequencing technology to identify miRNAs with similar expression patterns between fetal lung and lung tumours.

## Patients and methods

Eighty-nine pairs of tumour and adjacent non-malignant lung and five fetal lung tissue samples were profiled by miRNA-sequencing. Mann–Whitney U tests were performed with Benjamini-Hochberg multiple testing correction to identify common expression patterns.

## Results

We describe, for the first time, a comprehensive miRNA expression profile of human fetal lung. We have not only detected miRNAs that exhibit similar expression levels between fetal lung and non-malignant lung tissue which may play roles in lung tissue maintenance, but also identified multiple miRNAs with similar expression patterns between fetal lung and lung tumours. Some of these miRNAs have been previously implicated in lung cancer, while others have not yet been described to play a role in this disease. Pathways known to be targeted by miRNAs commonly expressed between fetal lung and lung tumours are involved in cell proliferation and angiogenesis. Further studies will elucidate the specific roles of these miRNAs in lung tumourigenesis.

## Conclusions

We identified several miRNAs with similar expression profiles between fetal lung and lung tumours which potentially play critical roles in lung cancer and might lead to the identification of promising therapeutic targets.

## Financial support

DDBS is supported by University of British Columbia 4YF scholarship, KLT by Vanier Canada Graduate Scholarship. This work was supported by grants from the Canadian Institutes for Health Research.

